# Supplementary material for: An Affordable phototherapy intensity meter using machine learning to improve the quality of care system for Hyperbilirubinemia in Indonesia
Source: PLoS One. 2023 May 2;18(5):e0285128. doi: 10.1371/journal.pone.0285128 (PMC10153703; doi:10.1371/journal.pone.0285128)
Supplement: Attachment — Submitted filename: Response to Reviewer.docx [file pone.0285128.s001.docx]

**Response to Reviewer**

Thank you for your feedback and suggestions on our submission. We would like to thank the reviewer for reading and commenting on our submission. We now have completed and addressed several comments stated in the revisions. Hopefully, revisions made on the manuscript would allow further considerations on indexing the article.

Reviewer #1: Whether this paper is suitable for this journal can not be judged by this reviewer. Beside that, this reviewer has only minor comments.

1)	If one reads the abstract only, the role of machine learning is unclear. It only becomes clear in the discussion that the goal is “calibration”. Please provide more information about the actual goal of ML in the Abstract and the Introduction.

Answer : We are very thankful for your comment regarding our manuscript. We now have added the information about ML in the abstract and introduction.

2)	Recently in 2022, AAP has revised their almost 20-30 year old guidelines. Please check whether this has an impact on the presented work.

Answer : We are very thankful for your reminder about the new version of AAP guidelines. We have checked, they still have a concern on the measurement intensity of phototherapy. We have renewed our references.

Reviewer #2: The problem of measuring light irradiance in phototherapy devices in low-income countries is important. The paper of Kristian et al. describes a new reliable phototherapy intensity meter which is very cheap in comparison to other meters. The comparison with Ohmeda Biliblanket Light Meter shows a 0.99 correlation score in all prototypes. The paper includes all data concerning the design of the prototype and the mathematical model to convert sensor readings to accurate measurements. The paper is interesting and, in my opinion, deserves publication.

Answer : We are very thankful for your comment regarding our manuscript.We hope that our manuscript can be beneficial for phototherapy practice especially in low-income countries.
